# Is it time to change the reference genome?

**DOI:** 10.1186/s13059-019-1774-4

**Published:** 2019-08-09

**Authors:** Sara Ballouz, Alexander Dobin, Jesse A. Gillis

**Affiliations:** Cold Spring Harbor Laboratory, The Stanley Institute for Cognitive Genomics, Cold Spring Harbor, NY 11724 USA

## Abstract

The use of the human reference genome has shaped methods and data across modern genomics. This has offered many benefits while creating a few constraints. In the following opinion, we outline the history, properties, and pitfalls of the current human reference genome. In a few illustrative analyses, we focus on its use for variant-calling, highlighting its nearness to a ‘type specimen’. We suggest that switching to a consensus reference would offer important advantages over the continued use of the current reference with few disadvantages.

## Why do we need references?

Until recently, a block of platinum-iridium in the International Bureau of Weights and Measures in France had a mass of precisely 1 kg. After 20 May 2019, the kilogram (Le Grand K) was redefined in reference to Planck’s constant (6.626070150 × 10^− 34^ kg∙m^2^/s [1]) and this will not change for the foreseeable future. The human genomic location of the tumor protein p53 is chromosome 17: 7,666,487–7,689,465 (genome reference GRCh38.p12). How permanent is the reference that determines this? We will never define the genome in terms of universal constants but can we do better than our current choice?

### Frame of reference

We need standards to communicate using a common frame of reference, but not all standards are created equal. If the platinum-iridium mass standard lost a few atoms, it would effectively change the measured mass of all other objects. It has always been clear that we would like to do better; the kilogram was the last SI unit still defined by a physical object. A reference defined with respect to a universal constant is not just more consistent, but also more accessible and practical. An arbitrary reference is, on the other hand, not very precisely shareable. Few people had access to the reference mass (there were six copies [2, 3]) and it was challenging to replicate (each copy had uniquely lost and gained atoms). Although a universal reference is the ideal, there are tradeoffs between utility, universality, and practicality that must be considered, in particular where no such universal constant is feasible.

### The burden of success

What would an ‘ideal’ reference genome look like? Because standards can take many forms, picking one is non-trivial. In practice, references can be a single sample or type, an average form or an empirical sampling, or a (universal) gold-standard (see Box 1 for definitions). One of the major intents behind the original sequencing of the human genome was to provide a tool for future analyses and this has been wildly successful. The current reference genome assembly works as the foundation for all genomic data and databases. It provides a scaffold for genome assembly, variant calling, RNA or other sequencing read alignment, gene annotation, and functional analysis. Genes are referred to by their loci, with their base positions defined by reference genome coordinates. Variants and alleles are labeled as such when compared to the reference (i.e., reference (REF) versus alternative (ALT)). Diploid and personal genomes are assembled using the reference as a scaffold, and RNA-seq reads are typically mapped to the reference genome.

These successes make the reference genome an essential resource in many research efforts. However, a few problems have arisen:

1. The reference genome is idiosyncratic. The data and assembly that made up the reference sequence reflect a highly specific process operating on highly specific samples. As such, the current reference can be thought of as a type specimen.
2. The reference genome is not a ‘healthy’ genome, ‘nor the most common, nor the longest, nor an ancestral haplotype’ [4]. Efforts to fix these ‘errors’ include adjusting alleles to the preferred or major allele [5, 6] or the use of targeted and ethnically matched genomes.
3. The reference genome is hard to re-evaluate. Using a reference of any type imposes some costs and some benefits. Different choices will be useful in different circumstances but these are very hard to establish when the choice of reference is largely arbitrary. If we pick a reference in a principled way, then those principles can also tell us when we should not pick the reference for our analyses.

In the following sections, we briefly address these three points by outlining the history of the human reference genome, demonstrating some of its important properties, and describing its utility in a variety of research ecosystems. Finally, we describe our version of a consensus genome and argue that it is a step in the right direction for future reference genome work. Our main interests are in defining the general principles and detailing the process of stepping in the right direction, even if the strides are small.

## The reference genome is idiosyncratic

### The history of the human reference genome

It is commonly said that we now live in the age of ‘Big Data’. In genomics, this refers to the hundreds of thousands of genomes sequenced from across all domains of life, with grand plans such as the Earth BioGenome Project (EBP) seeking to fill gaps in the coverage of eukaryotes [7]. The number of base pairs (bp) deposited in databases dedicated to sequencing data alone is at the peta scale (for example, the Sequence Read Archive database stands at around 2 × 10^16^ bp). The collection of sequencing data started humbly enough with the advent of Sanger sequencing in 1977. Having obtained the ability to read out the genome at base-pair resolution, researchers were able to access the genetic code of bacteriophages and their favorite genes. Why sequence the full human genome, or any genome for that matter? The first reason was the desire for ‘Big Science’ for biology [8]. Large projects existed in other fields such as physics, so why not in biology? If other species were being sequenced, then why not humans? Of course there were more pragmatic reasons for the suggestion. In addition to demonstrating technological feasibility, genome-scale science would enable comprehensive investigation of genetic differences both within and across species [9, 10]. In addition, sequencing an entire genome would allow the identification of all genes in a given species, and not only those that were the target of a monogenic disease (such as *HTT* in Huntington’s disease [11]) or of interest to a field (for example, *P53* in cancer [12]). The sequences of genomes would serve as useful toolboxes for probing unknown genomic regions, allowing the functional annotation of genes, the discovery of regulatory regions, and potentially the discovery of novel functional sequences. The Human Genome Project was conceived with these various desires in mind [13].

### The human reference assembly is continually being improved upon

The Human Genome Project was a gargantuan effort for its time, costing close to 3 billion US dollars to complete. The first draft genome was published in 2001 [14], along with the competing project from Celera [15]. The ‘complete’ genome, meaning 99% of the euchromatic sequence with multiple gaps in the assembly, was announced in 2003 [16]. Beyond launching the field of human genomics, the Human Genome Project also prompted the development of many of the principles behind public genomic data sharing, set out in the Bermuda Principles, that ensured that the reference genome was a public resource [17]. As a direct consequence, the use and improvement of the reference has made genomics a rapidly growing and evolving field. The first major discovery was the scale at which the human genome was littered with repetitive elements, making both sequencing hard and the assembly of the sequenced reads a computationally challenging problem [18]. In time, single-molecule technologies generating longer reads [19–21] and algorithmic advancements [22–24] have been used to improve the reference significantly. Currently, the human genome is at version 38 (GRCh38 [25]), which now has fewer than 1000 reported gaps, driven by the efforts of the Genome Research Consortium (GRC) [4, 26].

## The reference genome is not a baseline

### The current reference genome is a type specimen

**Fig. 1:**
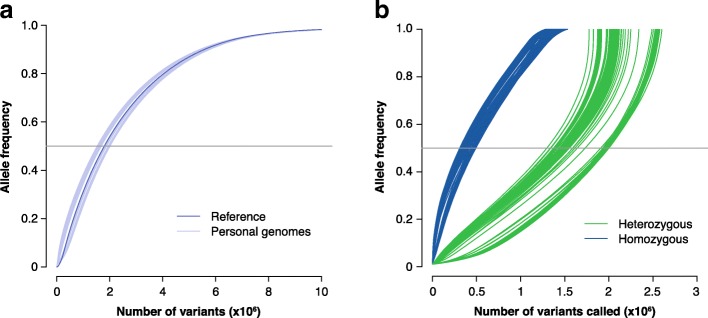
The reference genome is a type specimen. **a** Cumulative distributions of variants in the reference genome and those in personal/individual genomes. If we collapse the diploid whole genomes genotyped in the 1000 Genomes Project into haploid genomes, we can observe just how similar the reference is to an individual genome. First, taking population allele frequencies from a random sample of 100 individual genomes, we generated new haploid ‘reference’ sequences. We replaced the alleles of the reference genome with the personal homozygous variant, and a randomly chosen heterozygous allele. For simplicity, all calculations were performed against the autosomal chromosomes of the GRCh37 assembly and include only single nucleotide bi-allelic variants (i.e., only two alleles per single nucleotide polymorphism (SNP)). **b** Cumulative distributions of allele frequencies for variants called in 100 randomly chosen personal genomes, computed against the reference genome. Here, the presence of a variant with respect to the reference is quite likely to mean that the reference itself has the ‘variant’ with respect to any default expectation, particularly if the variant is homozygous

Although the reference genome is meant to be a standard, what that means in a practical sense is not clearly defined. For example, the allelic diversity within the reference genome is not an average of the global population (or any population), but rather contains long stretches that are highly specific to one individual. Of the 20 donors the reference was meant to sample from, 70% of the sequence was obtained from a single sample, ‘RPC-11’, from an individual who had a high risk for diabetes [27]. The remaining 30% is split 23% from 10 samples and 7% from over 50 sources [28]. After the sequencing of the first personal genomes in 2007 [29, 30], the emerging differences between genomes suggested that the reference could not easily serve as a universal or ‘gold-standard’ genome (see Box 1 for definitions). This observation is easily extended to other populations [31–34], where higher diversity can be observed. The HapMap project [35, 36] and the subsequent 1000 Genomes Project [37] were a partial consequence of the need to sample broader population variability [38]. Although the first major efforts to improve the reference focused on the need to fill in the gaps, work is now shifting towards incorporating diversity, through the addition of alternative loci scaffolds and haplotype sequences [39]. But just how similar to a personal genome is the current reference? We performed a short series of analyses to answer this question (Fig. 1), using the 1000 Genomes Project samples. Looking first at the allele frequencies (AF) of known variants, we found that around two million reference alleles have population frequencies of less than 0.5, indicating that they are the minor allele (dark blue line in Fig. 1a). This might seem high for a reference. In fact, the allelic distribution of the current reference is almost identical to the allelic distributions of personal genomes sampled from the 1000 Genomes Project (light blue lines in Fig. 1a). In practice, the current reference can be considered a well-defined (and well-assembled) haploid personal genome. As such, it is a good type specimen, exemplifying the properties of the individual genomes. This means, however, that the reference genome does not represent a default genome any more than any other arbitrarily chosen personal genome would.

### Reference bias

Because the reference genome is close to being a type specimen, it can distort results where it’s sequence is not very typical. In alignment, reference bias refers to the tendency for some reads or sequences to map more readily to the reference alleles, whereas reads with non-reference alleles may not be mapped or mapped at lower rates. In RNA-seq-based alignment and quantification, reference bias has a major impact when differential mapping matters (such as in allele-specific expression), but can be overcome by the use of personal genomes or through the filtering of biased sites [40–42]. In variant calling, reference bias can be more important. Alignment to the reference to infer variation related to disease is still a step in most analyses, and is crucial in clinical assignments of variant significance and interpretation [43, 44]. In these cases, reference bias will induce a particular error. Variant callers might call more ‘variants’ when the reference alleles are rare or could fail to call variants that are rare but also shared by the reference [45–48]. Owing to the presence of rare alleles in the reference genome**,** some known pathogenic variants are easily ignored as benign [25]. A variant called with respect to the reference genome will be biased, reflecting the properties of the reference genome rather than properties that are broadly shared in the population. Indeed, continuing with our analysis (Fig. 1b), if we compare the variant calls within personal genomes against the reference, we find that close to two-thirds of the homozygous variants (blue lines) and one-third of the heterozygous variants (green lines) actually have allele frequencies above 0.5. Variation with respect to the reference is quite likely to indicate the presence of a ‘variant’ in the reference genome with respect to any default expectation, particularly if that ‘variant’ is homozygous.

## The reference genome is hard to re-evaluate

### Type specimen references are often good enough

A research ecosystem has grown up around the reference and has mostly taken advantage of its virtues while compensating for its flaws. In alignment, for example, masked, enhanced, or diploid references have been used. The masking of repetitive regions or rare variants is a partial solution for improving the mapping and assembly of short reads. Enhanced and diploid genomes include additional alleles or sequences that are inserted into the current reference [47–55], helping to remove reference bias. In addition, because the reference genome is a collapsed diploid, work on purely homozygous genomes (termed platinum references) will provide true haploid genomes (such as that of the CHM1 cell line, which was derived from a molar pregnancy [56, 57]). More long-term fixes include the generation of new independent alternative references that eliminate the particularities of the original samples, such as those proposed by the McDonnell *Genome* Institute (MGI) Reference Genome Improvement project [58]. The goal there is to amend the lack of diversity of the reference by creating gold genomes: gold-standard references each specific for an individual population. Alongside these new standard genomes, personal or personalized genomes will become more common in clinical settings, with individuals’ own genomes (potentially from birth) being used throughout their lives for diagnostic assessments.

### Change is tricky

Any change to the current reference will require a large effort from the genomics field to adopt new practices. The most popular recommendation is the development of pan-genomes, comprising a collection of multiple genomes from the same species [59]. More complex than a single haploid reference sequence, a pan-genome contains all possible DNA sequences, many of which may be missing from any one individual [60]. A pan-genome can be represented as a directed graph [61], in which alternative paths stand in for both structural and single variants [62]. These are particularly useful for plants where ploidy exists within a species [63], or in bacteria where different strains have lost or gained genes [64]. Adopting the graph genome as a reference reflects not just the inclusion of additional data, but also the introduction of a novel data structure and format. Although graph genomes are well defined, their incorporation into existing research practice is not a trivial matter and tools to facilitate this are under active development [65–67]. A human pan-genome may improve variant calling by virtue of containing more variation [68], but this is offset by the difficulties in referring to such a reference. When compared with a linear reference genome, the coordinates in a pan-genome are harder to incorporate into existing software structures [69]. This is an issue because the current reference genome is the foundation of all genomics data. Variant databases use the reference coordinate systems, as do most gene and transcript annotations. Genome browsers use linear tracks of genomic data, and graph visualizations (e.g., cactus graphs [70]) are hard to interpret. Graph genomes have many properties to recommend them and are a potential future for genome references, but they will come at some cost and obtaining community buy-in may be particularly challenging.

## Seeking consensus

### Why a consensus?

Alongside personal genomes, major alleles have been useful in improving disease analysis and alignment [45], especially in regions of high variation (such as the human leukocyte antigen (HLA) locus) or for clinically relevant analyses where variant pathogenicity was misattributed (see examples in [48, 71]). In the same way that the consensus sequences of transcription-factor-binding motifs represent the most common version of the motif, a consensus genome represents the most common alleles and variants within a population. The adoption of a consensus genome would be comparatively painless to existing research practice, because the consensus would look substantially like a new reference in the current mode, but it would bring real improvements in interpretation and generalizability to new uses. Incorporating major alleles takes us half-way to a graph genome in terms of accuracy [72]. A consensus genome offers some benefits with almost no costs: (i) it is easy to replicate and accessible to evaluate anew from data; (ii) it is empirical with an explicit meaning to baseline (common); (iii) it is easily open to novel evaluation; and (iv) it can be recalculated whenever that is necessary to establish new baselines (e.g., for different populations).

We are not the first to suggest this or similar changes. For example, Dewey et al. [45] used major alleles in the sequence to study the HLA. Minor alleles (assessed in [71]) or those that are absent from certain ethnically distinct populations cause trouble in downstream clinical assessments [73] and tools have been built to screen for them [48]. The Locus Reference Genomic Project (LRG) is working to improve on gene sequences, primarily to correct for minor and disease alleles in variant significance assessments. A related gene-specific correction was first proposed by Balasubramanian et al. [74], who aimed to incorporate functional diversity in the protein-coding genome by using the ancestral allele. In this case, rather than using the most common or representative allele in a population, the variant alleles carried by the last common ancestor of all humans are incorporated into the sequence. Balasubramanian et al. [74] argued that this strategy provided an ethnically and population neutral version of a reference genome that is more stable (there is only one version) than the reference genomes recommended by others [75]. Its use is also limited, however, to positions in the genome for which information on the ancestral variant is available (including out-group sequence) and, practically speaking, a reference genome that was built in this way would be very similar to a re-weighted consensus across populations. More recently, a consensus-style genome was built from 1000 Genome Project alleles by Karthikeyan et al. [76] to improve on variant calling. These authors were able to eliminate 30% of false-positive calls and achieved an 8% improvement in true positives, despite using an older version of the reference (h19). A final major consideration is the inclusion of structural variants (SVs), which Audano et al. [77] described in recent work on a canonical human reference. The inclusion of SVs in the genome not only improves mapping accuracy, but also helps us to understand the impact of variants on protein function. An SV database, such as the recent gnomAD project release [78], will be key to the identification of best practices for their inclusion in a reference. Importantly, it is only now that we have enough genomes available that it is timely and feasible to generate a useful consensus genome [79, 80]. The key observation is not that one option is superior to any other, but that by specifying the population and the purpose of the analysis, the differences can be progressively lessened.

### What would a consensus genome look like?

**Fig. 2:**
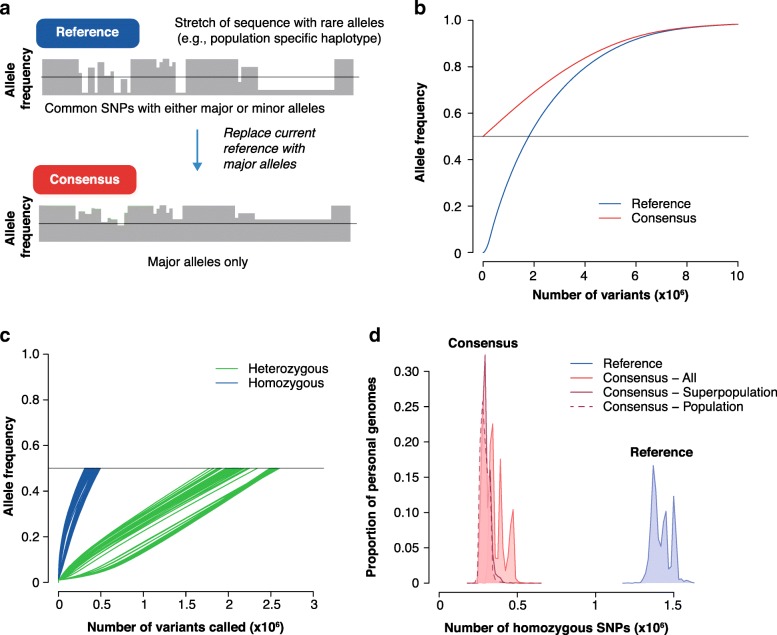
How consensus alleles improve the interpretability of the reference. **a** To build a consensus genome, we replaced minor alleles within the current reference with their major alleles (allele frequency (AF) > 0.5) across all bi-allelic SNPs. **b** Cumulative distributions of variants in the consensus genome (red line) and the current reference (blue line). **c** Cumulative distributions of AFs for variants in 100 randomly chosen personal genomes, computed against a consensus genome. **d** Distribution of the number of homozygous single nucleotide variants (SNVs) in 2504 personal genomes, computed against the reference, against an all-human consensus, the mean of the super-population consensuses and the mean of the population consensuses. The consensus reference for each of the five super-populations leads to an additional reduction in the number of homozygous variants in the personal genomes for each super-population (dark red curve). Further breakdown into 26 representative populations does not dramatically reduce the number of homozygous variants (dashed red line). Super-populations are defined broadly as: *AFR* African, *AMR* admixed American, *EAS* East Asian, *EUR* European, *SAS* South Asian

In the simplest of cases, a consensus genome remains a haploid linear reference, in which each base pair represents the most commonly observed allele in a population. As a parallel to our assessment in the previous section, we show this by looking at the variants called from the personal genomes sampled from the 1000 Genomes Project (Fig. 2). For illustrative purposes, we constructed a consensus genome by replacing all alleles with their major allele (Fig. 2a), as measured in the 1000 Genomes Project dataset. Repeating the previous analysis, we first note that the distribution of alleles are all above 0.5 as designed (Fig. 2b). Second, the personal variants that were called are all below the population frequencies of 0.5 as expected, and we see that the total number of variants called has been significantly reduced (Fig. 2c). Importantly, the number of homozygous variants called when using the consensus rather than the current reference is reduced from about 1.5 million to around 0.5 million. The distribution of the number of homozygous variants in all personal genomes in the 1000 Genomes Project collection against the standard reference (blue line) and consensus reference (red line) has shifted markedly (Fig. 2d).

In addition, the reference genome can stray far from the average not just randomly (because of the presence of minor alleles) but also systematically, reflecting variation drawn from a particular population. A recent pan-assembly of African genomes directly spoke to the necessity for population-specific references, because approximately 10% of DNA sequence (~ 300 Mbp) from these genomes was ‘missing’ from the GRCh38 reference [81]. Indigenous and minor populations are understudied in general, a shortcoming that will need to be remedied in order to provide adequate clinical and medical care to individuals from these populations [82]. For example, certain drugs will be more effective and safer in some populations than in others because the presence of certain variants will change drug metabolism. To expand on this and to test for population-specific impacts, we now build population-specific consensus genomes using the allele frequencies of the five major populations represented in the 1000 Genomes Project data. Population-specific consensus genomes display a modest reduction in the number of homozygous variants called (darker red lines in Fig. 2d), and a tightening of the spread of the distribution, as would be expected of a more refined null. This suggests that the modal peaks are population-specific variants, and that the use of population-typical data is helpful in these and related tasks.

### What would research built around a consensus genome look like?

**Fig. 3:**
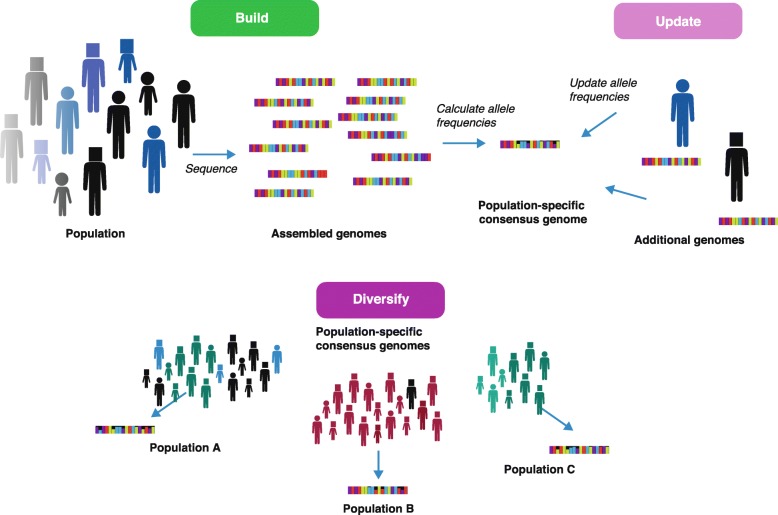
How-to reference. For future or new populations, sequencing is followed by building the consensus sequence from those genomes. Any new genomes will only adjust and improve on the current consensus on the basis of a change in allele frequencies. Finally, the reference can be replicated and diversified into other population-specific references

The ‘consensus’ that we describe in Fig. 2 uses both the existing reference and our knowledge of population allele frequencies. This is particularly straightforward for single nucleotide polymorphisms (SNPs), but more complex genomic rearrangements can also be iteratively incorporated into a consensus genome. Practically speaking, any novel variant is called with respect to an existing reference, and once that variant is known to be common, it becomes part of the new consensus. Relatively few genomes are necessary to ascertain that a novel variant is the major allele, making the iterative improvement of the reference a community-based effort, and one that can be tailored to suit different purposes. For example, even though the major allele consensus reference will not typically preserve the long-range association between variants, this association can be imposed as a specific constraint by picking consensus sequences at larger scales (i.e., using haplotype blocks). We think that explicit choices of alternative references, particularly population-specific ones, will be a natural extension of the framework that we describe (Fig. 3), helping to reduce bias against underrepresented populations.

The importance of population and individual diversity mean that any choice of human reference needs to be carefully considered. In contrast to an inbred model organism such as the C57BL/6 mouse, where the reference is the gold standard, the human reference is not of fixed utility and individual differences from it can be hard to interpret. As population datasets become broader and individual datasets become deeper, it appears to be time to think about both the virtues of the current reference and our potential options to replace or augment it. The switch to a consensus genome would not be a transformational change to current practice and would provide a far from perfect standard, but because it would offer incremental, broad-based, and progressive improvement, we believe that it is time to make this change.

Box 1 Definitions: what we talk about when we talk about genomes **Alternate (ALT) allele.** The non-reference allele.

**Ancestral genome.** A version of the reference genome in which each position is represented by the ancestral allele. An ancestral allele is defined as the allele shared by the most common ancestor.

**Baseline genome.** A minimum or starting point to compare against. This is not necessarily the ‘best-performing’.

**Consensus genome.** A version of the reference genome in which each position represents the most common base in a specified population. Other terms for this include the **null, empirical**, or **canonical** genome.

**Diploid**. An organism or cell with a double set of chromosomes, so that each position is represented by two genes or alleles.

**Genotype.** The genetic makeup of an organism.

**Graph genome.** A non-linear representation of a genome, in which paths in the graph represent individual genomes.

**Haploid.** An organism or cell with a single set of chromosomes.

**Haplotype.** An inherited series of genetic elements.

**Normal genome**. A disease-free genome, or a genome with only typical disease risk. The latter use is context dependent and thus hard to define in absolute or genetic terms.

**Pan-genome.** A collection of multiple genomes from a single species. These are usually represented in graph form.

**Personal genome.** A single individual’s diploid genome sequence or assembly.

**Platinum genome.** A purely haploid but complete genome sequence, usually derived from hydatidiform moles or molar pregnancies. Molar pregnancies are abnormal pregnancies that occur when a sperm has fertilized an oocyte that has no genome, and the

subsequent divisions result in cells with diploid genomes that are derived from a single

paternal genome.

**Reference allele.** The allele that is present in the reference genome (REF).

**Reference genome/assembly.** A linear representation of the genome of a species. Most assemblies are haploid, although some loci are represented more than once in alternate scaffolds. For humans, the reference genome assembly was generated from multiple individuals. It does not represent a single haplotype, nor the ancestral haplotype.

**Type specimen.** The reference sample used to define the general class by example, often for a species.

**Universal/gold-standard genome**. A reference genome that is the best-performing for a specified purpose or, if ‘universal’, any likely purpose.

**Variant.** A difference from the reference or standard sequence (i.e., polymorphic sites). Variants include single-nucleotide polymorphisms (SNPs or SNVs) and structural deletions or insertions (indels). They can also encompass much larger chromosomal rearrangements (translocations, duplications, or deletions) that result in copy-number variants (CNVs).

## Abbreviation

HLA: Human leukocyte antigen
